# Heavy alcohol consumption before and after negative life events in late mid-life: longitudinal latent trajectory analyses

**DOI:** 10.1136/jech-2021-217204

**Published:** 2021-09-23

**Authors:** Neda Agahi, Lucas Morin, Marianna Virtanen, Jaana Pentti, Johan Fritzell, Jussi Vahtera, Sari Stenholm

**Affiliations:** 1 Aging Research Center, Karolinska Institute/Stockholm University, Solna, Sweden; 2 Department of Medical Epidemiology and Biostatistics, Karolinska Institute, Solna, Sweden; 3 Inserm CIC 1431, University Hospital of Besançon, Besançon, France; 4 School of Educational Sciences and Psychology, University of Eastern Finland, Joensuu, Finland; 5 Division of Insurance Medicine, Karolinska Institute, Solna, Sweden; 6 Department of Public Health, University of Turku and Turku University Hospital, Turku, Finland; 7 Centre for Population Health Research, University of Turku and Turku University Hospital, Turku, Finland; 8 Clinicum, Faculty of Medicine, University of Helsinki, Helsinki, Finland

**Keywords:** alcoholism, behaviour, addictive, longitudinal studies, substance abuse

## Abstract

**Background:**

People who experience negative life events report more heavy alcohol consumption compared with people without these experiences, but little is known about patterns of change *within* this group. This study aims to identify trajectories of heavy alcohol consumption before and after experiencing either divorce, or severe illness or death in the family. Furthermore, the aim is to examine characteristics of individuals belonging to each trajectory.

**Methods:**

Longitudinal study of public sector employees from the Finnish Retirement and Aging Study with up to 5 years of annual follow-ups (n=6783; eligible sample n=1393). Divorce and severe illness or death in the family represented negative life events. Heavy alcohol consumption was categorised as >14 units/week.

**Results:**

Based on latent trajectory analysis, three trajectories of heavy drinking were identified both for divorce and for severe illness or death in the family: ‘No heavy drinking’ (82% illness/death, 75% divorce), ‘Constant heavy drinking’ (10% illness/death, 13% divorce) and ‘Decreasing heavy drinking’ (7% illness/death, 12% divorce). Constant heavy drinkers surrounding illness or death in the family were more likely to be men, report depression and anxiety and to smoke than those with no heavy drinking. Constant heavy drinkers surrounding divorce were also more likely to be men and to report depression compared with those with no heavy drinking.

**Conclusions:**

Most older workers who experience divorce or severe illness or death in the family have stable drinking patterns regarding heavy alcohol consumption, that is, most do not initiate or stop heavy drinking.

## Introduction

Negative life events entail elevated stress levels and usually imply disruptions to daily life and habits, including changes in behaviours such as alcohol consumption.^1 2^ Increased alcohol consumption can serve as a way of coping with the stress and reducing tension,^3 4^ even at older ages.^5^ However, the association between negative life events and alcohol consumption is complex. Earlier studies have found both increasing and decreasing alcohol consumption in response to, and in anticipation of, various life events such as divorce, severe illness in the family and widowhood.^2 6^

The relationship between divorce and alcohol consumption is bidirectional and multifaceted, partly because risky alcohol use can be both a driver and a consequence of divorce. Previous studies have suggested that heavy drinking increases the risk of divorce,^7 8^ but that, in turn, divorcing increases the risk of subsequent heavy drinking^2 9^ and alcohol use disorder.^10^ On the other hand, heavy drinking has also been found to decrease before divorce.^2^ It may also decrease afterwards, particularly if divorce is perceived as a relief. These patterns may reflect psychological distress preceding or following divorce, which have been found to be different among women and men.^11^

Severe illness of a spouse or other family member is another source of stress^12^ and the death of a loved one is typically one of the most challenging stressful events that individuals encounter throughout their life course. Severe illness in the family is stressful not only because of uncertainty regarding disease progression, but also because of the burden of social support and caregiving that is placed on family members.^13^ Experiencing social and emotional burden as a family caregiver comes with a higher probability of risky alcohol use.^14^ Studies investigating the loss of a spouse or other loved person have shown that there are various responses with regard to heavy alcohol consumption surrounding the event. For example, Tamers and colleagues^2^ found a decrease in heavy consumption at the time of spousal loss, and among women also during the period preceding the loss. In contrast, prior to the death of other loved persons than a spouse, heavy alcohol consumption increased, but then started decreasing at the time of or after the death.^2^

Taken together, previous findings suggest that people who experience negative life events are more likely to have heavy alcohol consumption habits compared with people who do not. However, less is known about various patterns of heavy drinking *within* the group of people who experience negative life events. Previous studies that have followed individuals through the life events, with both pre-measurements and post-measurements, have focused on the changes in mean levels of alcohol consumption,^2^ but a more detailed description of the various trajectories of heavy alcohol consumption surrounding negative life events is lacking. The aim of this study was twofold. First, to identify and differentiate the most common trajectories of heavy alcohol consumption before and after experiencing a divorce or severe illness or death of a family member. Second, to examine the characteristics of individuals belonging to each of the identified trajectories, with a focus on sociodemographic, work, health and behavioural characteristics.

## Methods

### Study population and design

The Finnish Retirement and Aging Study (FIREA) is an ongoing cohort established in 2013. The eligible population included all public sector employees whose individual retirement date was between 2014 and 2019 and who worked in the year 2012 in 1 of 27 municipalities in southwest Finland or in 9 selected cities or 5 hospital districts around Finland (n=10 629).^15^ Participants were contacted 18 months prior to their estimated retirement date, which was obtained from the pension insurance institute for the municipal sector in Finland (Keva), by sending a questionnaire, which was thereafter sent annually, at least four times in total.

By the end of 2019, 6783 participants had responded to at least one survey. For our purposes, the study population was limited to participants who reported a negative life event (264 for divorce, 1129 for severe illness or death in the family) in any of the surveys. Participants who reported their alcohol consumption in both the survey before and after the event were included in the analytical sample (154 for divorce, 622 for severe illness or death in the family). To allow comparison with those who did not experience these life events, we derived a so-called ‘control group’ that consisted of FIREA participants who did not report divorce or severe illness or death in the family and who reported their alcohol consumption in two consecutive surveys to resemble the eligibility criteria for the other groups (n=3354); see flow chart in online supplemental figure S1.

10.1136/jech-2021-217204.supp1 Supplementary data

**Figure 1:**
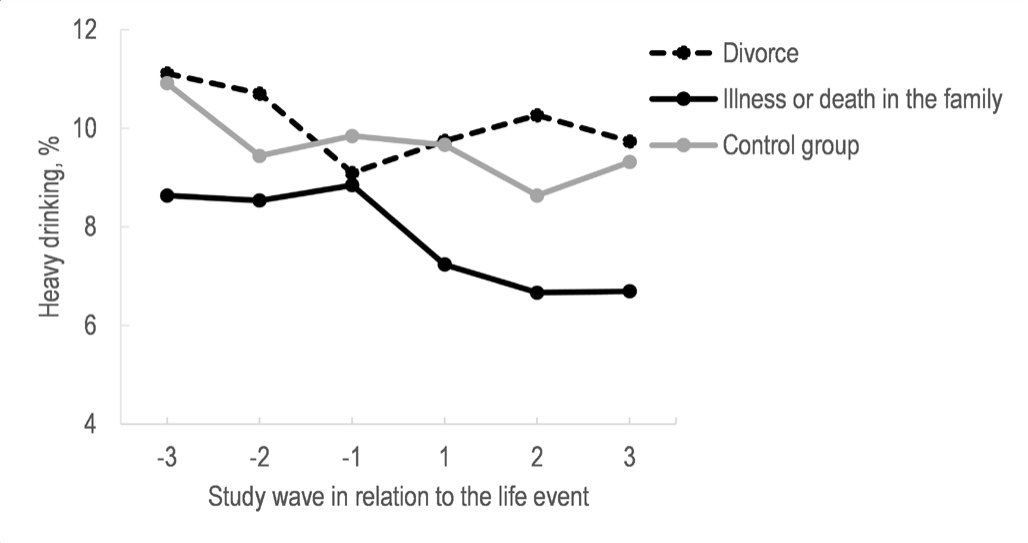
Proportion of heavy drinking at different time points among those with a severe illness or death in the family, and divorce, and among those without these life events (control group).

For each individual respondent, we centred the data around the first life event (divorce, illness, death) that occurred. There were three possible study waves before each life event (−3, –2, −1), and three possible waves after (+1, +2, +3). Each participant may have taken part in maximum six study waves. On average, participants provided information on alcohol consumption at 3.9 (SD=0.8) study waves for the event of illness, 4.0 (SD=0.9) for death and 3.9 (SD=0.9) for divorce. The difference between each study wave was on average 1 year. For those in the ‘control group’, there was no life event, but a mock event year was randomly assigned for each participant enabling to organise the data in a similar manner as in the divorce and severe illness or death in the family groups.

### Variables

#### Negative interpersonal life events

Negative life events within the past 12 months were self-reported through a structured questionnaire that included 12 items. We used two types of negative life events: (1) severe illness in the family (spouse/child) and/or death in the family (spouse/child), and (2) divorce.

#### Heavy alcohol consumption

Participants reported their habitual frequency and amount of beer, wine and spirits consumption, in weekly units of alcohol. One unit corresponds to 12 g of pure alcohol, or the equivalent of 33 cL of beer, 12 cL of wine, or 4 cL of spirits.^16^ We defined heavy alcohol consumption as weekly consumption exceeding 14 units for both women and men according to current UK guidelines.^17^

#### Sociodemographic, work, health and behavioural characteristics

Characteristics previously shown to be associated with heavy alcohol consumption or negative life events were included as covariates. Information was taken from the last questionnaire before the life event of interest. Information about the participants’ date of birth, sex and occupational title was obtained from Keva. The main indicator of socioeconomic status (SES) was occupational status. Occupational titles were coded according to the International Standard Classification of Occupations (ISCO) and categorised into three groups: high (ISCO classes 1–2, for example, teachers, physicians), intermediate (ISCO classes 3–4, for example, registered nurses, technicians), and low (ISCO classes 5–9, for example, cleaners, maintenance workers). Another socioeconomic indicator was neighbourhood socioeconomic disadvantage, based on neighbourhood characteristics from Statistics Finland Grid Databases. The socioeconomic composition for each grid (250×250 m) included average annual income of households, mean number of years of education of residents above 18 years and proportion unemployed among adult residents in the labour force.^18^ For each of the three variables, a standardised z-score was derived based on the total Finnish population (mean=0, SD=1). A summary score for neighbourhood SES was calculated by taking the mean value across the three z-scores. Lower summary scores indicate lower neighbourhood disadvantage. Residential mobility data, based on a complete history of residential addresses with latitude and longitude coordinates, were obtained from the Population Register Center for each participant. Using open-source Geographical Information Systems (http://www.qgis.org/en/site/), data on the cumulative residential neighbourhood disadvantage for each time point were linked to the participants’ home addresses by the latitude and longitude coordinates. A cumulative socioeconomic disadvantage score weighted by residential time at each location was calculated for each participant. The summary score of neighbourhood socioeconomic disadvantage was dichotomised into low (≤0) and high (>0), in accordance with earlier studies.^19^ Marital status was categorised into married/cohabiting versus not, and work status into full-time work, part-time work and retired. History of doctor-diagnosed depression was based on the question, ‘Has your doctor ever told you that you have or have had…?’, and depression was one condition in a list of 26 diseases.

Anxiety was measured with the short six-item form of the State-Trait Anxiety Inventory,^20^ which included statements of how one generally feels (‘I feel… calm/tense/upset/relaxed/content/worried’). The response scale was 1 (not at all), 2 (little bit), 3 (quite much), 4 (very much). We calculated the mean of the items and midpoint was used to categorise into high anxiety (≥2.5) versus low anxiety (<2.5).^21^

The number of social network ties was assessed using the social convoy model,^22^ which is based on a set of three circles representing different levels of closeness to the respondent. The respondent is asked to name individuals belonging to each circle. Total number of network ties was determined by summing up the number of persons in all three circles, and then categorised into 0–10 or 11 persons or more. This approach has been used in earlier studies.^23 24^ Smoking was dichotomised into current smoking versus former and never-smoking.

### Statistical analysis

We used complete case analysis. Prevalence of heavy drinking in each study wave was calculated separately for those experiencing divorce, severe illness or death in the family, as well as for those not experiencing these life events. To illustrate changes and heterogeneity in heavy drinking in the years preceding and following the specific life events, we used latent trajectory analysis, a family of statistical methods used to identify distinctive groups of individuals who show similar developmental trajectories over time.^25^ Separate analyses were conducted for (1) illness or death in the family and (2) divorce. We used the PROC TRAJ program to estimate latent trajectories in the statistical software SAS V.9.4 (SAS Institute) and Nagin’s two-step procedure to determine the optimal number of trajectories and choose the number and order of regression parameters.^25^ First, we fitted increasing number of trajectory models with cubic polynomial shape for heavy alcohol consumption until no improvement in model fit was observed. Both linear and quadratic models were tested. Assessment of model fit was based on Bayesian Information Criterion values, Akaike Information Criterion values, log-likelihood and posterior probabilities. Model fit statistics are presented in online supplemental table S1. For both illness/death in the family and divorce, three-trajectory solutions with the best fit were selected.

10.1136/jech-2021-217204.supp2 Supplementary data

To examine the characteristics of individuals in the different trajectory groups, we used multinomial logistic regression analyses. The reference group was the ‘No heavy drinking’ group for both life events. Analyses were adjusted for age and gender.

### Results

Table 1 shows the characteristics of study participants who experienced severe illness or death in the family or divorce from the wave before the event, as well as for those not experiencing these life events. Overall, the three groups were similar. The group that experienced illness or death in the family were slightly older and more often women, married/cohabiting and have a depression diagnosis. The group that experienced divorce had also more often a depression diagnosis and elevated anxiety and current smoking.

**Table 1:** Characteristics of specific life event populations in the study wave before the event and in a ‘control group’ of individuals who did not experience these life events

	‘Control group’ (N=3354)	Illness or death in the family (N=622)		Divorce (N=154)	
			P value		P value
Age in years, mean (SD)	63.6 (1.5)	63.8 (1.5)	0.005	63.7 (1.6)	0.369
Gender, no (%)			0.019		0.606
Men	599 (17.9)	87 (14.0)		25 (16.2)	
Women	2755 (82.1)	535 (86.0)		129 (83.8)	
Marital status, no (%)			<0.0001		
Married/cohabiting	2328 (71.3)	499 (81.9)		–	
Not married/cohabiting	937 (28.7)	110 (18.1)		–	
Missing	89	13		–	
Occupational status, no (%)			0.763		0.170
High	1122 (33.7)	199 (32.3)		41 (26.8)	
Intermediate	999 (30.0)	187 (30.3)		54 (35.3)	
Low	1207 (36.3)	231 (37.4)		58 (37.9)	
Missing	26	5		1	
Neighbourhood disadvantage, no (%)			0.373		0.196
Low	2064 (65.5)	389 (67.4)		83 (60.1)	
High	1087 (34.5)	188 (32.6)		55 (39.9)	
Missing	203	45		16	
Work status, no (%)			0.209		0.944
Full-time work	1454 (43.8)	252 (40.8)		66 (43.1)	
Part-time work	397 (11.9)	66 (10.7)		17 (11.1)	
Retired	1493 (44.7)	299 (48.5)		70 (45.8)	
Missing	10	5		1	
Depression diagnosis, no (%)			0.023		0.034
No	2581 (85.2)	461 (81.5)		106 (78.5)	
Yes	448 (14.8)	105 (18.5)		29 (21.5)	
Missing	325	56		19	
Anxiety, no (%)			0.287		0.078
Low	3010 (91.6)	554 (90.2)		132 (87.4)	
High	278 (8.5)	60 (9.8)		19 (12.6)	
Missing	68	8		3	
Social network size, no (%)			0.163		0.504
>10	2832 (85.5)	539 (87.6)		127 (83.5)	
≤10	480 (14.5)	76 (12.4)		25 (16.5)	
Missing	42	7		2	
Alcohol risk use, no (%)			0.547		0.781
No	2959 (88.2)	554 (89.1)		137 (89.0)	
Yes	395 (11.8)	68 (10.9)		17 (11.0)	
Missing					
Smoking, no (%)			0.324		0.053
Never or former	2988 (90.8)	547 (89.5)		130 (86.1)	
Current	303 (9.2)	64 (10.5)		21 (13.9)	
Missing	63	11		3	

P values indicate differences between the life event populations and the ‘control group’.

Figure 1 shows proportions of heavy drinking at different time points among those with a severe illness or death in the family, and divorce, and among those without these life events. Those who experienced severe illness or death in the family showed on average a declining trend in heavy drinking after the event, whereas those who experienced divorce had increasing proportions of heavy drinking after the event. Among those without these life events, the proportion of heavy drinking was more stable, with a slight decrease, over the follow-up time.

**Figure 2:**
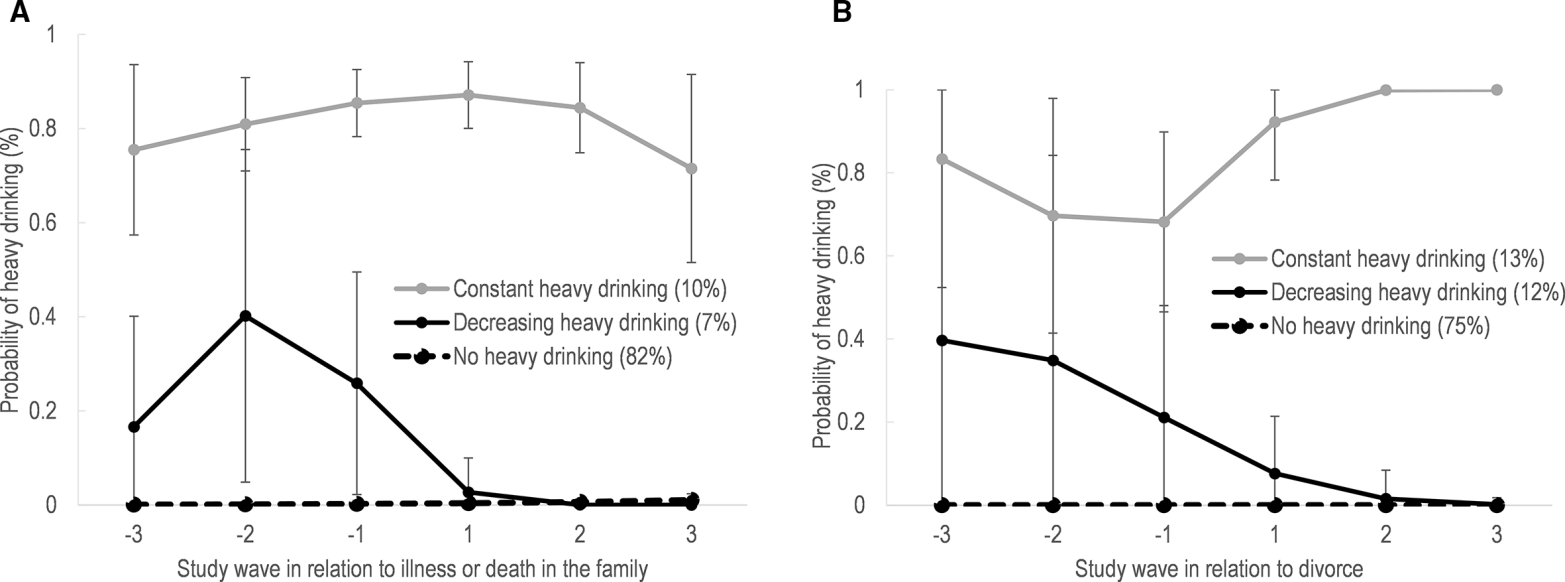
Trajectories of heavy drinking before and after (A) severe illness or death in the family, and (B) divorce.

Figure 2 illustrates the trajectories of heavy alcohol consumption surrounding severe illness or death in the family and divorce. The largest trajectory group for both life events was the ‘No heavy drinking’ group. Among participants who experienced severe illness or death in the family, 82% were in the ‘No heavy drinking’ group. The other two trajectories were ‘Decreasing heavy drinking’ showing elevated probability of heavy drinking before the illness or death in the family, followed by a decrease surrounding the event and low after (7%), and ‘Constant heavy drinking’ showing constantly high probability of heavy drinking (10%). Among participants who experienced a divorce, 75% belonged to the ‘No heavy drinking’ trajectory. The other two trajectories were one with elevated probability of heavy drinking before the divorce which then decreased, ‘Decreasing heavy drinking’ (12%), and one with constantly high heavy drinking throughout the period with an increase after divorce, ‘Constant heavy drinking’ (13%).

Tables 2 and 3 (and online supplemental tables S2 and S3) show the characteristics of the individuals belonging to each of the trajectories surrounding the two life events. Table 2 and online supplemental table S2 show that among those experiencing severe illness and/or death in the family, people in the ‘Constant heavy drinking’ trajectory were more often men, reported history of depression, had high levels of anxiety and were current smokers compared with those in the ‘No heavy drinking’ trajectory group. The ‘Decreasing heavy drinking’ trajectory did not differ significantly from the ‘No heavy drinking’ trajectory, but ORs and proportions indicate that they were retired or worked part-time and smoked to a higher extent.

10.1136/jech-2021-217204.supp3 Supplementary data

10.1136/jech-2021-217204.supp4 Supplementary data

**Table 2:** Association between individual characteristics and heavy drinking trajectories among people who experienced severe illness or death in the family

	No heavy drinking	Decreasing heavy drinking		Constant heavy drinking	
	OR (ref)	OR	95% CI	OR	95% CI
Male vs female	1	0.78	0.18 to 3.42	**5.43**	**3.05 to 9.66**
Married/cohabiting: no vs yes	1	0.59	0.17 to 2.02	1.56	0.80 to 3.02
Intermediate vs high occupational status	1	0.53	0.20 to 1.43	0.58	0.30 to 1.11
Low vs high occupational status	1	0.64	0.22 to 1.82	0.73	0.37 to 1.43
High vs low neighbourhood disadvantage	1	0.99	0.39 to 2.51	0.98	0.54 to 1.78
Part-time work and retired vs full-time	1	2.15	0.76 to 6.14	0.84	0.46 to 1.51
Depression: yes vs no	1	0.47	0.11 to 2.05	**2.44**	**1.30 to 4.60**
Anxiety: yes vs no	1	1.05	0.24 to 4.66	**2.33**	**1.09 to 4.95**
Social network size: ≤10 vs >10	1	0.71	0.16 to 3.11	0.72	0.30 to 1.72
Smoking: yes vs no	1	2.22	0.72 to 6.83	**3.15**	**1.55 to 6.38**

Multinomial logistic regression models adjusted for gender and age.

Bold text indicates statistically significant findings.

**Table 3:** Association between individual characteristics and heavy drinking trajectories among people who experienced a divorce

	No heavy drinking	Decreasing heavy drinking		Constant heavy drinking	
	OR (ref)	OR	95% CI	OR	95% CI
Male vs female	1	**5.37**	**1.33 to 21.65**	**4.25**	**1.45 to 12.47**
Intermediate vs high occupational status	1	0.77	0.16 to 3.57	0.44	0.14 to 1.42
Low vs high occupational status	1	0.44	0.07 to 2.76	0.32	0.09 to 1.19
High vs low neighbourhood disadvantage	1	1.23	0.29 to 5.17	0.28	0.08 to 1.04
Part-time work and retired vs full-time	1	2.36	0.42 to 13.09	0.64	0.22 to 1.86
Depression: yes vs no	1	4.43	0.86 to 22.80	**4.66**	**1.53 to 14.20**
Anxiety: yes vs no	1	1.82	0.33 to 10.06	1.79	0.49 to 6.47
Social network size: ≤10 vs >10	1	1.20	0.22 to 6.43	1.71	0.53 to 5.50
Smoking: yes vs no	1	--		3.08	0.96 to 9.91

Multinomial logistic regression models adjusted for gender and age.

Bold text indicates statistically significant findings.

Table 3 and online supplemental table S3 describe characteristics of people in the trajectory groups surrounding divorce. Compared with the ‘No heavy drinking’ trajectory, those belonging to the ‘Decreasing heavy drinking’ and ‘Constant heavy drinking’ trajectories were men to a higher extent and reported more depression (statistically significant only for the constant trajectory). The ‘Constant heavy drinking’ group also had higher occupational status, lived in areas with low neighbourhood deprivation and were current smokers to a higher extent, but again differences did not reach statistical significance.

### Discussion

This study investigated trajectories of heavy alcohol consumption surrounding negative interpersonal life events among older individuals working in the municipal sector: severe illness or death of a family member and divorce. Overall, results suggest that for most working people in their 60s going through these life events, there is often no change in drinking behaviours with regard to initiating or giving up heavy alcohol consumption. A vast majority (75%–82%) reported no heavy alcohol consumption neither before nor after the event in question. Similarly, about one-tenth reported heavy drinking both before and after divorce or illness/death in the family.

An important finding, however, was that for both types of life events, one trajectory indicated elevated probability of heavy drinking 2–3 years before the event, but then decreasing. This may be indicative of an anticipatory effect, in that the likelihood of heavy drinking is elevated prior to the event, perhaps because of elevated stress levels or anticipatory grief.^26^ Anticipatory effects prior to negative life events have also been suggested in other studies regarding health deterioration prior to spousal loss,^27^ and increased heavy drinking prior to losing a loved one (non-spouse).^2^ In the case of divorce, this result is in line with previous findings suggesting that heavy drinking often precedes divorce,^7 8^ but also that heavy drinking starts decreasing already prior to the divorce.^2^

We found some indications of gender differences. Previous studies suggest that consequences of losing family members are potentially stronger among women than men,^28^ but that men tended to use alcohol as a means to cope to a larger extent.^29^ Our results showed that men were over-represented in both ‘Constant heavy drinking’ trajectory and the ‘Decreasing heavy drinking’ trajectory surrounding divorce, compared with the ‘No heavy drinking’ trajectory. This may suggest that men use alcohol to cope to a larger extent than women, but it may also simply be that men generally drink more than women. Further studies with higher representation of men in the study sample are needed to investigate trajectories for women and men separately.

Furthermore, results showed that for both life events, those belonging to the ‘Constant heavy drinking’ group were more likely to be men, reported more depression and had higher occupational status (not statistically significant) compared with the ‘No heavy drinking’ trajectory. Similar characteristics have been reported in other studies.^30^ A possible explanation is that men and individuals in higher socioeconomic groups in these age groups drink more alcohol in general^31^ and thus also when faced with negative life events. The higher probability of depression (and anxiety for the group experiencing illness/death in the family) in the ‘Constant heavy drinking’ group confirms the comorbidity of substance use and depressive/anxiety disorders.^32^ Thus, it seems important to pay special attention to people who report depressive symptoms and undergo negative life events and find ways to prevent excessive alcohol use.

Several strengths and limitations need to be considered when interpreting the findings. A major strength is the annual data collections that allow detailed descriptions of change surrounding the life event. Another strength is that the respondents, due to the narrow age span (60–65 years), are rather homogeneous regarding the life phase they are in. Furthermore, we used the current UK guidelines for defining heavy drinking,^17^ which simplifies comparison across studies. However, since drinking habits and patterns differ between countries, for example, Finland having relatively high alcohol-attributable death rates,^33^ people in different countries may also have different response and coping strategies for negative life events, and thus direct comparison with other countries is challenging.

Limitation to consider when interpreting these results is, for example, the limited sample size in some trajectory groups (primarily for divorce, which is relatively uncommon in this age group), leading to statistical uncertainty and wide CIs, which may restrict the generalisability of our findings. Another limitation that may restrict generalisability and oversimplify reality is that the identification of a certain number of distinct alcohol trajectories ‘forces’ all individuals in the sample into one of these trajectories. Previous studies on alcohol trajectories have suggested that trajectory groups may not be as distinct as the models suggest.^34 35^ The reliance on self-reported alcohol consumption represents another important limitation, as we may underestimate actual amounts consumed. Since we investigate individual-level changes, our assumption is that this underestimation is similar over time and therefore less of a problem for our study purposes. In addition, the dichotomisation of the outcome into heavy alcohol consumption versus not may mask variations in drinking over time within both categories. For example, people who are categorised as having a heavy alcohol consumption (>14 units per week) may increase their weekly amount of drinking without it being captured in our analyses. Regarding the measurement of life events, we view illness and death in the family as well as divorce as negative life events, but we do not have a measure of how they were perceived by the participants. Further studies are warranted where the perceived stress and hardship surrounding the events are taken into consideration. Furthermore, non-response is likely higher surrounding life events and consequently people who have experienced a life event and are included in our analytical sample (ie, have taken part in the surveys just before and after the event in question) may differ from those who have not experienced these life events. Based on the results presented in table 1, it seems that the only consistent difference between those who have experienced a life event and those who have not among the variables that we have investigated is the history of a doctor-diagnosed depression. Finally, as the study sample consisted of people in their 60s who were still active in the work force, it is likely a relatively healthy sample compared with those aged 60–65 years old in general. In addition, the over-representation of women means that the relative size of the trajectory groups indicating heavy drinking may be underestimated as compared with the general population in Finland.

In conclusion, our findings suggest that most people who experience divorce or severe illness or death in the family in their 60s have unchanged drinking patterns when it comes to heavy alcohol consumption. In other words, people tend to keep their heavy, or non-heavy, alcohol consumption habits when going through these life events, although increases or decreases *within* the categories may occur. Depression was more common in the ‘Constant heavy drinking’ group, suggesting that comorbidity between substance use and depressive/anxiety disorder may add to the vulnerability of this group.

**What is already known on this subject**

- People who experience severe illness or death in the family or divorce are more likely to report heavy alcohol consumption compared with people without these experiences.
- Little is known about various patterns of heavy drinking before and after these life events within the group of people who experience them.

**What this study adds**

- For most working people in their 60s going through severe illness or death in the family or divorce, there was no change in drinking behaviours regarding initiating or giving up heavy drinking.
- A vast majority reported no heavy drinking neither before nor after the event, but about one-tenth reported heavy drinking both before and after, and another one-tenth had elevated probability of heavy drinking before the event, but then decreasing.
- Trajectories of heavy drinking were more common among men.

## Data Availability

Data are available upon reasonable request.
