# Supplementary figures and images for: Heavy alcohol consumption before and after negative life events in late mid-life: longitudinal latent trajectory analyses
Source: J Epidemiol Community Health. 2021 Sep 23;76(4):360–6. doi: 10.1136/jech-2021-217204 (PMC8921586; doi:10.1136/jech-2021-217204)

Supplementary Figure S1. Flow-chart for study sample.

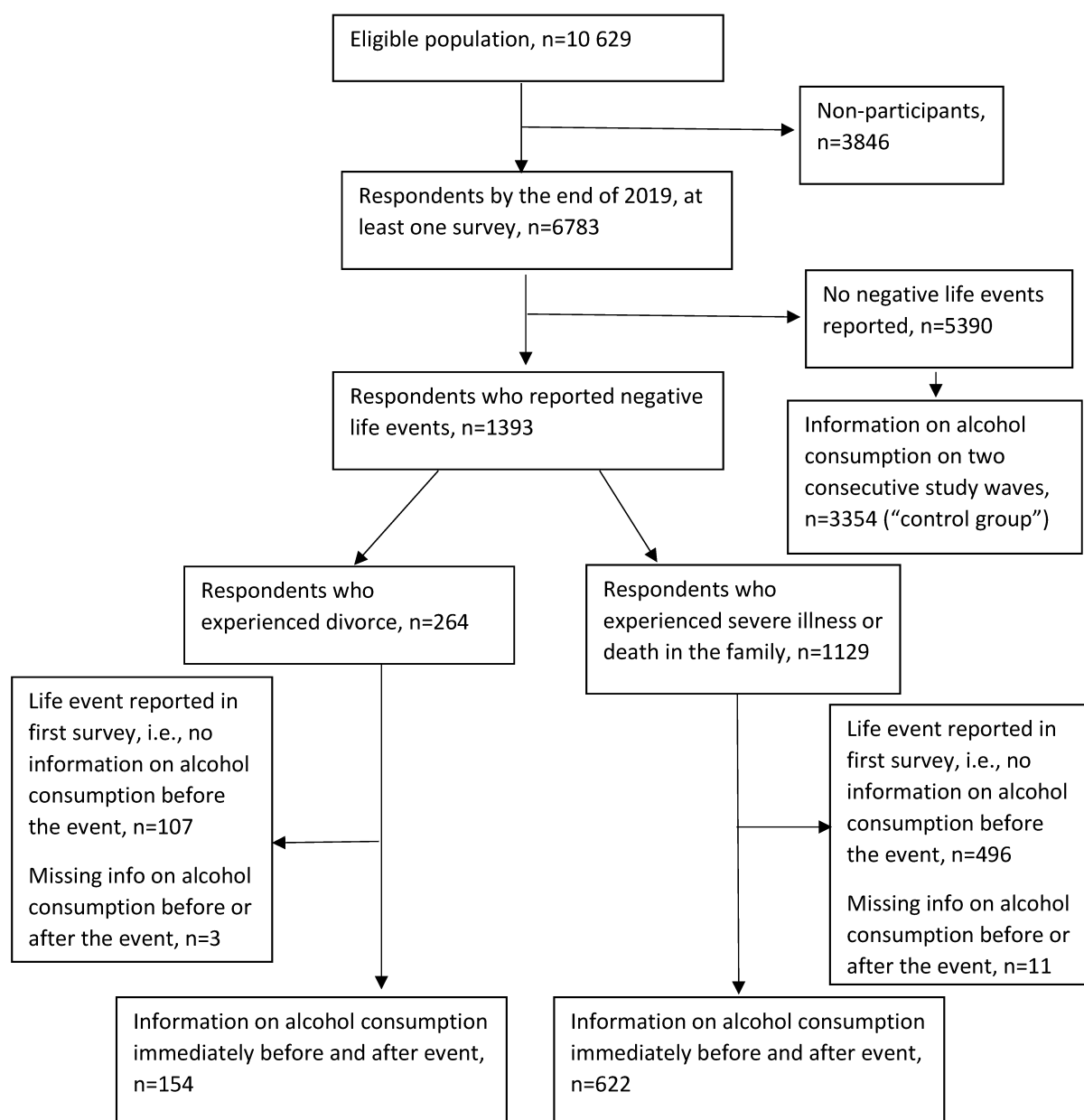

Supplement: Supplementary data [file jech-2021-217204supp001.pdf]
