# Supplementary material for: Heavy alcohol consumption before and after negative life events in late mid-life: longitudinal latent trajectory analyses
Source: J Epidemiol Community Health. 2021 Sep 23;76(4):360–6. doi: 10.1136/jech-2021-217204 (PMC8921586; doi:10.1136/jech-2021-217204)
Supplement: Supplementary data [file jech-2021-217204supp002.pdf]

SUPPLEMENTARY TABLES AND FIGURES

Supplementary Table S1. Model fit statistics of the latent trajectory analysis from polynomial models with 1 to 3 trajectories for different life events.

Number of trajectories	Polynomial order*	BIC†	AIC	Log-likelihood	Average posterior probabilities	Smallest group (%)
Illness or death in the family						
1	3	-792.73	-783.87	-779.87	1	100
2	33	-498.67	-478.72	-469.72	0.996/0.975	11.3
3	333	-512.28	-481.25	-467.25	0.985/0.865/0.963	2.7
3	133	-504.23	-477.63	-465.63	0.956/0.819/0.985	7
**3**	**123**	**-501.35**	**-467.97**	**-465.97**	**0.954/0.826/0.977**	**7.2**
Divorce						
1	3	-235.96	-229.89	-225.89	1	100
2	33	-159.36	-145.69	-136.69	0.993/0.961	15
3	333	-170.1	-148.84	-134.84	0.938/0.955/0.955	8.6
3	133	-165.58	-147.36	-135.36	0.980/0.709/0.968	3.7
**3**	**123**	**-162.79**	**-146.09**	**-135.09**	**0.926/0.899/0.980**	**12**

Notes: BIC = Bayesian Information Criterion values; AIC = Akaike Information Criterion values. *Polynomial function 1 refers to linear, 2 to quadratic and 3 to cubic shape of trajectory. The selected trajectory solutions are bolded. † BIC value is based on the number of individuals.
