# Supplementary material for: Heavy alcohol consumption before and after negative life events in late mid-life: longitudinal latent trajectory analyses
Source: J Epidemiol Community Health. 2021 Sep 23;76(4):360–6. doi: 10.1136/jech-2021-217204 (PMC8921586; doi:10.1136/jech-2021-217204)
Supplement: Supplementary data [file jech-2021-217204supp003.pdf]

Supplementary Table S2. Characteristics of individuals belonging to the three trajectories of heavy drinking among those experiencing illness or death in the family.

	**No heavy drinking (n=535)**	**Decreasing heavy drinking (n=24)**	**Constant heavy drinking (n=63)**
Age in years, mean (SD)	63.7 (1.5)	64.3 (1.2)	63.9 (1.7)
Gender, %			
Men	69.0	2.3	28.7
Women	88.8	4.1	7.1
Marital status, %			
Married / cohabiting	86.2	4.2	9.6
Not married / cohabiting	84.6	2.7	12.7
Occupational status, %			
High	80.9	5.0	14.1
Intermediate	87.7	3.2	9.1
Low	89.2	3.0	7.8
Neighborhood disadvantage, %			
Low	85.6	3.6	10.8
High	86.7	3.7	9.6
Work status, %			
Full-time work	87.3	2.0	10.7
Part-time work or retired	85.2	5.2	9.6
Depression diagnosis, %			
No	87.0	4.6	8.5
Yes	81.0	1.9	17.1
Anxiety, %			
Low	86.8	4.0	9.2
High	78.3	3.3	18.3
Social network size, %			
> 10	85.7	4.1	10.2
≤ 10	88.2	2.6	9.2
Smoking, %			
Never or former	87.4	3.7	9.0
Current	71.9	6.3	21.9
