# Supplementary material for: Heavy alcohol consumption before and after negative life events in late mid-life: longitudinal latent trajectory analyses
Source: J Epidemiol Community Health. 2021 Sep 23;76(4):360–6. doi: 10.1136/jech-2021-217204 (PMC8921586; doi:10.1136/jech-2021-217204)
Supplement: Supplementary data [file jech-2021-217204supp004.pdf]

Supplementary Table S3. Characteristics of individuals belonging to the three trajectories of heavy drinking among those experiencing a divorce.

	**No heavy drinking (n=124)**	**Decreasing heavy drinking (n=10)**	**Constant heavy drinking (n=20)**
Age in years, mean (SD)	63.6 (1.5)	64.2 (1.4)	63.7 (1.9)
Gender, %			
Men	56.0	16.0	28.0
Women	85.3	4.7	10.1
Occupational status, %			
High	68.3	9.8	22.0
Intermediate	88.9	3.7	7.4
Low	82.8	6.9	10.3
Neighborhood disadvantage, %			
Low	75.9	6.0	18.1
High	87.3	7.3	5.5
Work status, %			
Full-time work	81.8	3.0	15.2
Part-time work or retired	79.3	9.2	11.5
Depression diagnosis, %			
No	84.9	4.7	10.4
Yes	62.1	10.3	27.6
Anxiety, %			
Low	81.8	6.1	12.1
High	68.4	10.5	21.1
Social network size, %			
> 10	81.9	6.3	11.8
≤ 10	72.0	8.0	20.0
Smoking, %			
Never or former	81.5	7.7	10.8
Current	71.4	0.0	28.6
